# Hyperbaric oxygen therapy attenuates urethral stricture formation after urethral injury: an experimental rabbit study

**DOI:** 10.1007/s00345-026-06323-2

**Published:** 2026-03-04

**Authors:** Hasan Danış, Alpaslan Yüksel, Dursun Baba, Yusuf Şenoğlu, Arda Taşkın Taşkıran, Ekrem Başaran, Ali Tekin, Sinem Kantarcıoğlu Coşkun

**Affiliations:** 1 Department of Urology, Duzce Ataturk State Hospital, Duzce, Türkiye; 2 Department of Urology, Medeniyet University Prof. Dr. Süleyman Yalçın Hospital, İstanbul, Türkiye; 3 Department of Urology, Duzce University School of Medicine, Duzce, Türkiye; 4 Department of Urology, Marmara University School of Medicine, Istanbul, Türkiye; 5 Department of Urology, Acıbadem University Atakent Hospital, İstanbul, Türkiye; 6 Department of Pathology, T.C. Ministry of Health, Ballkesir Provincial Health Directorate, Atatürk City Hospital, Balikesir, Türkiye

**Keywords:** Urethral stricture, Hyperbaric oxygenation, Experimental rabbit model, Fibrosis

## Abstract

**Purpose:**

Urethral stricture (US) is a frequent and challenging urological entity characterized by spongiofibrosis and luminal narrowing, often leading to recurrent infections, urinary retention, and upper tract deterioration. Despite various endoscopic and open surgical options, high recurrence rates remain a major problem, and there is no established medical therapy to prevent stricture formation. To investigate the protective effect of hyperbaric oxygen (HBO) therapy on fibrosis and stricture formation in an experimental urethral injury model in rabbits using objective endoscopic, radiologic, and histopathologic parameters.

**Methods:**

Twenty-eight male New Zealand rabbits were randomized into three groups: sham (*n* = 8), urethral injury without treatment (control, *n* = 10), and urethral injury plus HBO (HBO, *n* = 10). Among the 20 rabbits with urethral injury, allocation to the untreated control group (Group 2) and the HBO treatment group (Group 3) was performed in a 1:1 ratio using a computer-generated randomization list. Standardized circumferential electrocoagulation was applied to the bulbar urethra in the control and HBO groups. HBO was administered at 2 ATA, 90 min/day for 21 consecutive days starting on the day of injury. On day 28, all animals underwent urethroscopy and retrograde urethrography; urethral specimens were then harvested for histopathological evaluation. Fibrosis was graded (0–3) using Masson trichrome staining.

**Results:**

Normal urethral diameters were similar among the groups (*p* = 0.604). Median stricture diameter was significantly lower in the untreated control group compared with the HBO and sham groups (2.15 mm vs. 8.05 mm and normal segment, respectively; *p* < 0.001). Median percentage urethral narrowing was 78.52% in the control group and 23.51% in the HBO group (*p* < 0.001). Fibrosis scores were significantly higher in the control group than in the HBO and sham groups (*p* < 0.001).

**Conclusion:**

HBO therapy significantly attenuated fibrosis and urethral narrowing in this experimental urethral injury model. These findings suggest that HBO may represent a promising adjuvant strategy to prevent US formation after urethral trauma, warranting further clinical investigation.

## Purpose

Urethral stricture (US) is a pathology characterised by fibrotic scar formation developing in the urethral segment, often involving the corpus spongiosum. The clinical picture is characterised by narrowing of the urethral lumen and significant impairment of voiding functions, which are associated with this fibrotic process [1, 2]. Untreated US can result in severe complications, including prostatitis, epididymitis-orchitis, bladder stones, hydronephrosis, periurethral abscess, recurrent urinary tract infections, sepsis, and chronic renal failure due to prolonged retention [2].

The aetiology of US varies depending on the location of the stricture, the patient’s age, and previous interventions. However, it most commonly arises due to inflammatory, iatrogenic, traumatic, and idiopathic causes [3, 4].

The primary treatment modalities for US include dilation, internal urethrotomy, laser urethrotomy, urethral stenting, and open surgical reconstruction. Despite the fact that open surgeries achieve a high success rate of 85–90 per cent in the long term, endoscopic methods are generally preferred as the first-line treatment due to the more invasive nature and higher cost of open surgery [3, 4]. Nevertheless, the long-term success rates of endoscopic urethrotomy procedures remain low at 20–30%, which increases the need for repeat interventions and highlights the necessity to explore new, highly effective alternative treatments.

Hyperbaric oxygen therapy (HBO) constitutes an effective treatment modality entailing the inhalation of 100% oxygen at pressures in excess of 1 ATA (Atmosphere Absolute) within a closed pressure chamber. The present study investigates the current applications of the drug, which include the treatment of a wide range of medical conditions, including cases of CO poisoning and necrotising soft tissue infections [5, 6]. It has been established that HBO exerts a favourable influence on the various phases of wound healing, encompassing inflammation, proliferation and maturation. In addition, it has been demonstrated to provide substantial support to tissue healing through its antibacterial, antioxidant and oedema-reducing properties [7, 8].

In view of the aforementioned information, the objective of the present study was to evaluate the protective and restorative potential of HBO therapy, a non-invasive treatment which is increasingly utilised, on the development of US, a significant health problem in the present day. This was achieved by employing the positive biological effects of HBO therapy throughout all stages of wound healing, using objective histopathological and radiological parameters.

## Methodology

The present study was conducted at the Düzce University Faculty of Medicine Laboratory Animals Application and Research Centre (DUDAM) in accordance with international standards for the care of laboratory animals. The study was conducted with the permission of the Düzce University Faculty of Medicine Local Ethics Committee for Laboratory Animals, dated 19 February 2019 and numbered 2019/1/10. The study utilised a total of 28 male New Zealand rabbits, aged 4 months and weighing 2.5 ± 0.5 kg. The animals were housed under controlled laboratory conditions with a temperature of 23 °C, a humidity level of 50–60%, and a 12:12 h light-dark cycle. The animals were housed in separate cages throughout the study period and had unlimited access to standard laboratory feed and water. The present study was reported in accordance with the ARRIVE guidelines and the experimental animal ethics committee.

### Anesthesia, analgesia, and postoperative monitoring

After arrival at the laboratory, all rabbits were allowed to acclimatize to the environment for approximately one week without any procedures being performed. On the day of the intervention, all animals were weighed, and general anesthesia was induced by intramuscular injection of ketamine hydrochloride (15 mg/kg) and xylazine (6 mg/kg). All procedures were performed under this general anesthesia regimen. Following the procedure, appropriate postoperative analgesia was provided in accordance with institutional animal care guidelines, using meloxicam at a dose of 0.1 mg/kg once daily. Animals were monitored at least once daily throughout the study period for body weight, food and water intake, activity, grooming behavior, and signs of pain or distress. If any evidence of discomfort or distress was observed, additional analgesia was administered as required.

### Humane endpoints and euthanasia criteria

Humane endpoints were predefined in accordance with the institutional animal care and use committee (IACUC) guidelines. Criteria for early euthanasia included a loss of more than 15–20% of baseline body weight, persistent anorexia or severe dehydration, marked lethargy or immobility, labored breathing, or any other signs of unrelieved pain or distress despite analgesic treatment. Animals meeting these criteria were humanely euthanized under deep anesthesia using an overdose of ketamine, in accordance with the approved protocol. One rabbit that showed progressive clinical deterioration on day 26 was closely monitored and euthanized according to these humane endpoint criteria.

### Formation of experimental groups

This experiment was planned in accordance with the ARRIVE guidelines. The animals were divided into three groups: Group 1 (sham, *n* = 8), Group 2 (urethral injury without treatment, *n* = 10), and Group 3 (urethral injury plus HBO, *n* = 10). After induction of urethral injury, the 20 rabbits were randomly assigned to either the untreated control group (Group 2, *n* = 10) or the HBO treatment group (Group 3, *n* = 10) in a 1:1 ratio. Randomization was performed by an investigator not involved in the procedures using a computer-generated random number sequence (Microsoft Excel^®^), and group allocation was concealed in sequentially numbered, opaque, sealed envelopes that were opened immediately before the intervention. A formal a priori sample size calculation (power analysis) was not performed; the number of animals per group was determined based on previous experimental urethral stricture models in the literature and the 3R (replacement, reduction, refinement) principles, to balance ethical constraints with the need to obtain interpretable data. Baseline mean body weight was comparable among the three groups, with no statistically significant differences at the start of the experiment.

Group 1 (Sham, *n* = 8): No surgical procedures or treatments were administered to the subjects in this group. A 28-day follow-up period was conducted, after which urethroscopy and retrograde urethrography were performed to evaluate normal urethral anatomy. Following the completion of the procedures, the animals were euthanised and penectomy was performed for histopathological examination.

Group 2 (Untreated Control, *n* = 10): These were selected at random from 20 rabbits with induced urethral injury. No treatment was administered to the animals in this group. One rabbit exhibited clinical deterioration on postoperative day 26 and was euthanised following essential endoscopic and radiological evaluations. The rabbit was included in the study. No complications were observed in the other nine rabbits, and they were euthanised after imaging on postoperative day 28.

Group 3 (HBO Treatment Group, *n* = 10): Animals were selected at random from those with induced urethral injury. Commencing on the day on which the injury was induced, hyperbaric oxygen therapy was administered once daily for a period of 21 days at 2 ATA pressure for a duration of 90 min. The animals were placed in triple cages in a specially manufactured hyperbaric oxygen chamber. No serious complications were observed during the treatment process. Only three animals experienced mild urethral bleeding during the second week, which resolved spontaneously the following day. All animals were euthanised on postoperative day 28, following imaging procedures.

### Urethral injury model

**Fig. 1:**
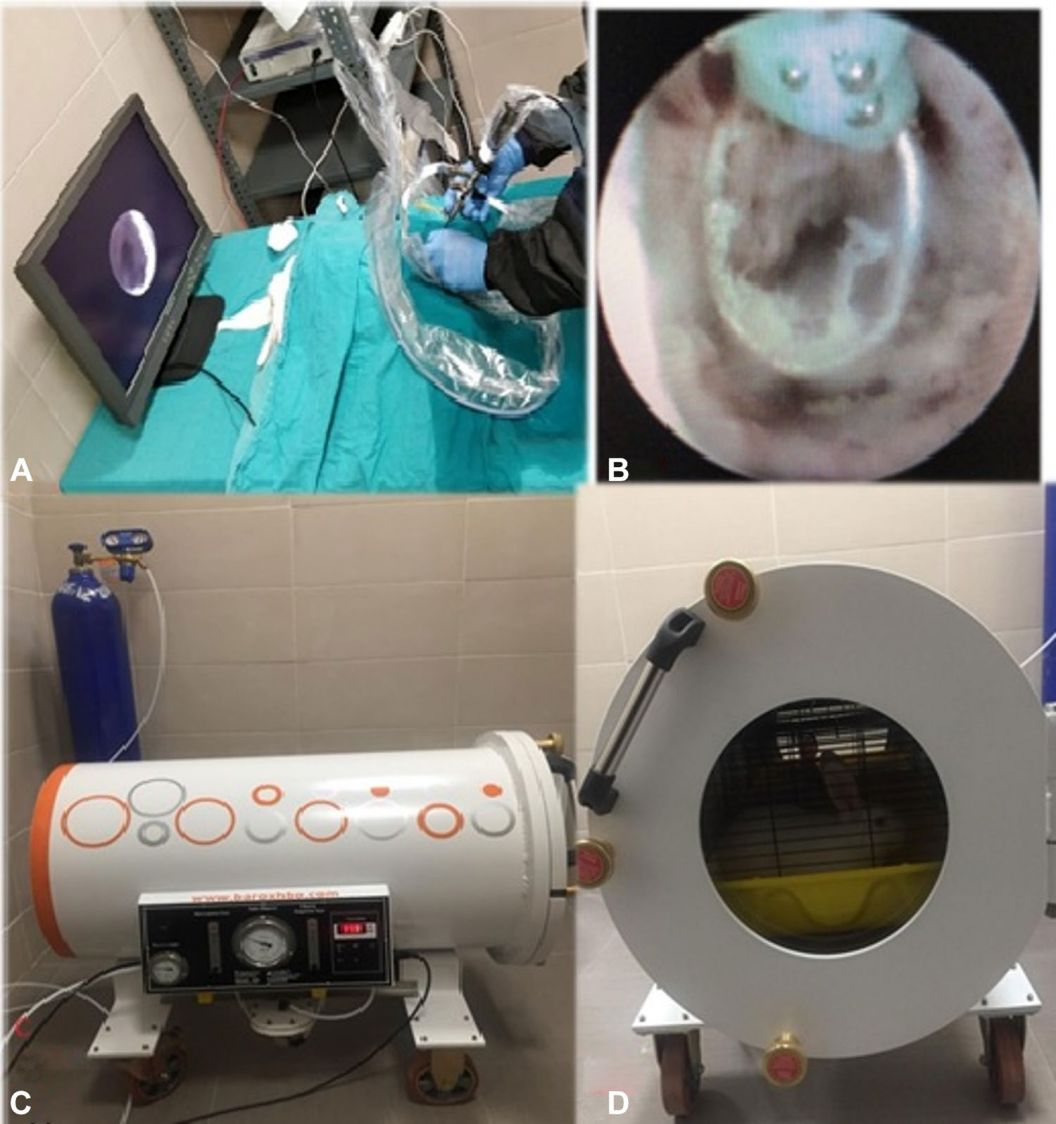
**A** Experimental procedure image, **B** Application of cauterisation at the mid-urethral level using an electrosurgical unit at 40 W power, **C** Hyperbaric oxygen chamber specially designed for experimental animals. **D** View of rabbits inside the chamber

The animals were sedated and placed in a supine position, after which the genital area was prepared in an aseptic manner using povidone-iodine. The 11 F paediatric resectoscope (Karl Storz) was inserted through the external meatus, and a 10 mm circumferential mucosal injury was created at the level of the middle urethra, distal to the external sphincter, using a 40 W paediatric electrocautery device (Fig. 1A and B). The cauterisation process was continued until the observation of mucosal whitening and ulceration [9, 10]. All procedures were performed by the same surgeon to ensure standardisation. The 28-day follow-up period was selected as it represents the phase where acute inflammation typically resolves and is replaced by established collagen deposition and tissue remodeling in the rabbit urethra, allowing for an objective assessment of permanent stricture formation.

### Hyperbaric oxygen therapy protocol

HBO therapy was initiated on the same day postoperatively, and a total of 21 sessions were administered. The treatment was performed in a specially designed HBO chamber measuring 100 × 55 cm, large enough to accommodate three rabbit cages (Fig. 1C and D).

Session protocol: Washing the chamber with 100% oxygen for 5 min, reaching 2 ATA pressure in 10 min, applying treatment at 2 ATA for 60 min, ending the session with a 15-minute decompression. Each session lasted a total of 90 min.

### Outcome measures

The primary and secondary endpoints were predefined to objectively evaluate the therapeutic impact of HBO therapy on urethral healing and the prevention of stricture formation. The primary endpoint of the study was the percentage of urethral narrowing, as determined by diameter measurements obtained from retrograde urethrography on postoperative day 28. Secondary endpoints included the endoscopic grading of urethral stricture severity using a pediatric cystoscope and the histopathological fibrosis score (0–3) on Masson’s trichrome–stained sections to quantify submucosal collagen accumulation.

### Endoscopic and radiological evaluation

Urethroscopy and retrograde urethrography were performed on all animals on postoperative day 28 to evaluate morphological changes in the urethra. In animals with strictures, the relevant segment was carefully measured and recorded.

### Histopathological examination

Following the sacrifice of the animals, the removal of all urethral tissues was conducted. In the groups with strictures, the relevant area was excised 1 cm distally and proximally beyond the lesion margins. All samples were preserved in 10% formaldehyde for a 24-hour period. Following the processing of the tissue samples, paraffin blocks were prepared and serial sections measuring 5 μm in thickness were obtained using a microtome. Sections were subjected to staining with haematoxylin-eosin (H&E) and Masson’s trichrome, and evaluated under a light microscope at 8×, 20×, 40× and 100× magnification.

Fibrosis grading was performed by a single pathologist on Masson’s trichrome–stained sections using a 4-point scale (0–3) as follows: 0, no fibrosis; 1, mild fibrosis (< 25% of the tissue); 2, moderate fibrosis (25–50%); and 3, severe fibrosis (> 50%) [11]. All histopathological evaluations were conducted by an experienced genitourinary pathologist who was blinded to group allocation and treatment.

### Statistical analysis

The data were subjected to descriptive statistics, which included the calculation of the mean, standard deviation, and minimum–maximum values. The distribution suitability of continuous variables was assessed using the Shapiro–Wilk test. The homogeneity of variance was examined using the Levene test. In the context of group comparisons, the application of statistical tests such as the Independent Samples t-test, Kruskal-Wallis test and Mann–Whitney U test was contingent upon the satisfaction of specific assumptions. Continuous variables are presented as mean ± standard deviation for normally distributed data, and as median and interquartile range (IQR) for non-normally distributed data, in order to ensure the most accurate representation of central tendency according to the distribution characteristics of each parameter. All analyses were performed using SPSS 22 software, and a p-value of less than 0.05 was considered to indicate statistical significance.

## Results

### Outcome measures

The primary endpoint of the study was the percentage urethral narrowing measured on retrograde urethrography. Secondary endpoints included endoscopic grading of urethral stricture severity and the histopathological fibrosis score (0–3) on Masson trichrome–stained sections.

### Endoscopic results

**Fig. 2:**
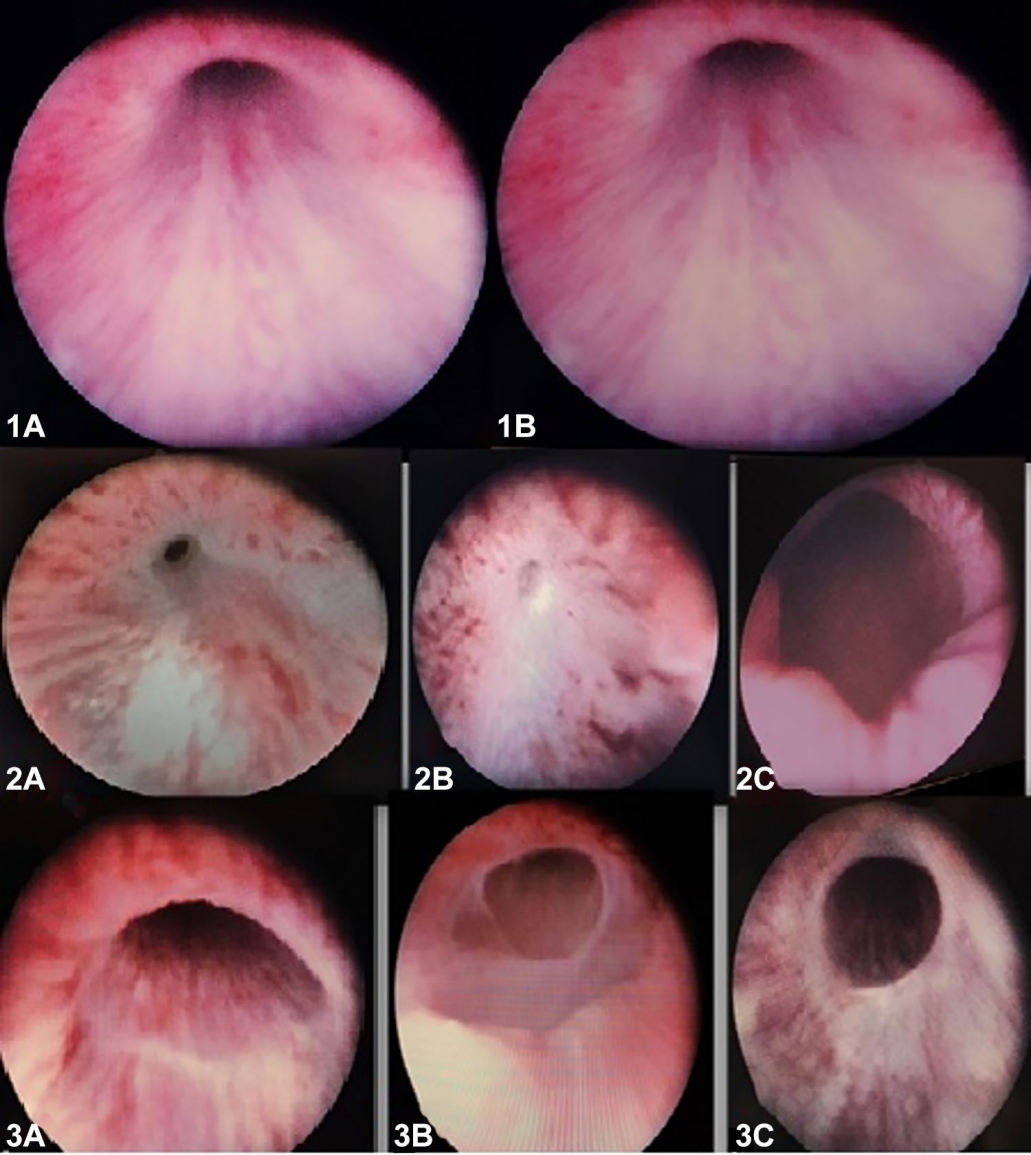
The following observations were made during the examination of the rabbits’ urethras using endoscopy: (1) The normal appearance of the urethra in Group 1 (sham) is illustrated in Fig. 1A and B. (2) The following section presents the endoscopic images of Group 2. The rabbit was diagnosed with moderate-to-severe urethral stricture (2A, 2B) and partial urethral stricture (2C). (3) The following section presents the endoscopic images of the treatment group: The classification system delineates three distinct categories: (3A) complete healing, (3B) mild stricture, and (3C) moderate stricture

At the conclusion of the 28-day follow-up period, the urethras of all rabbits were subjected to endoscopic evaluation using a paediatric cystoscope, and any morphological changes were meticulously documented. In Group 1 (the sham group), the urethral mucosa appeared normal in all eight rabbits examined, with no pathological findings or strictures detected (Fig. 2).

In Group 2, which received no treatment, strictures developed in all rabbits with induced urethral injury (Fig. 2). In the case of one rabbit, a stricture segment was observed that only partially narrowed the lumen. In contrast, the remaining animals exhibited areas of stricture that resulted in a closure of the lumen to a moderate to severe degree, yet still permitted urine passage. A subsequent endoscopic examination of the rabbit on postoperative day 26 revealed near-complete closure of the urethral lumen. Retrograde urethrography of the same rabbit showed minimal contrast passage, and laparotomy after sacrifice revealed widespread fluid accumulation in the abdomen and bladder perforation (Fig. 2).

In Group 3, which received HBO therapy, endoscopic examination revealed that the urethral mucosa was close to normal in three rabbits, partial stricture was present in three rabbits, and moderate stricture was present in four rabbits (Fig. 2). It was observed that none of the rabbits in this group exhibited a severe stricture area that resulted in complete narrowing of the lumen.

### Urethrographic results

**Fig. 3:**
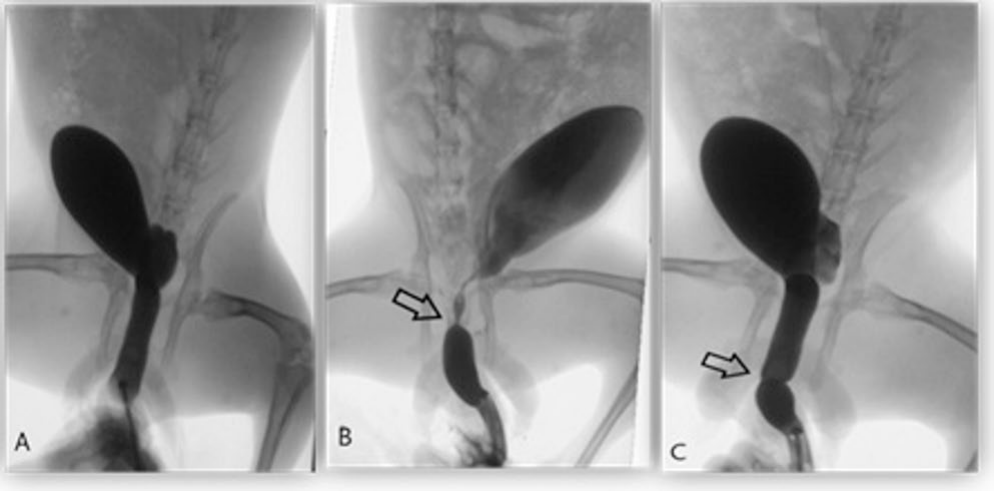
Urethral radiographic images in rabbits in Group 1, Group 2 and Group 3 A- Normal urethral appearance in rabbits in Group 1 B- Narrowing formation allowing minimal urine passage in Group 2, the untreated group. C- Urethrographic appearance of the nearly completely open urethra in Group 3, which received HBO therapy

Retrograde urethrography was performed on all rabbits on day 28 under anaesthesia. The urethral lumen was visualised by filling it with contrast medium (Fig. 3). The diameter of the urethra 1 cm distal to the stricture segment was accepted as the normal reference. No statistically significant difference was identified between the groups with regard to normal urethral diameters (Group 1: 10.76 mm; Group 2: 11.00 mm; Group 3: 10.32 mm; *p* = 0.604).

The mean urethral diameter was measured at 2.15 mm in Group 2, which did not receive treatment, and 8.05 mm in Group 3, which received HBO therapy. The stricture diameter in Group 2 was found to be significantly lower in comparison to Group 3 and the sham group (*p* < 0.001) (Table 1 ).

The evaluation of the percentage of urethral stricture revealed that the median stricture rate was 78.52% in Group 2, which did not receive treatment, and 23.51% in the HBO treatment group. The percentage of stricture in the group that did not receive treatment was significantly higher than that in the HBO group (*p* < 0.001) (Table 1).

**Table 1:** Radiological and histopathological comparison of urethral stricture severity among groups

Parameter	Group 1 (Sham)	Group 2 (no treatment)	Group 3 (HBO)	*p*
Normal urethral diameter, mm	10.76 ± 0.93 (9.20–12.10)	11.00 ± 0.96 (9.70–12.90)	10.32 ± 0.84 (9.10–11.70)	0.604
Urethral stricture diameter, mm	10.85 (1.15)	2.15 (0.90)	8.05 (1.90)	< 0.001
Percentage urethral narrowing, %	0.00 (–)	78.52 (7.78)	23.51 (11.99)	< 0.001
Pathological fibrosis score (0–3)	0 (0–0)	2.5 (2–3)	1 (1–2)	< 0.001

Data for normal urethral diameter are expressed as mean ± standard deviation (SD) with minimum–maximum range due to their normal distribution. Urethral diameter at the target level, percentage urethral narrowing, and pathological fibrosis scores (0–3 scale) are presented as median and interquartile range (IQR) as these variables followed a non-normal distribution. The choice between mean and median was determined based on the normality of data distribution (Shapiro–Wilk test) to ensure statistical accuracy. p values were calculated using one-way ANOVA for normally distributed variables and Kruskal–Wallis with post hoc Mann–Whitney U tests for non-normally distributed variables, as appropriate

HBO, hyperbaric oxygen; SD, standard deviation; IQR, interquartile range

### Histopathological results

Tissue samples from the urethra of the sacrificed animals were examined using Haematoxylin-Eosin and Masson’s Trichrome stains. These stains were used in accordance with standard tissue processing procedures. The evaluation of fibrosis through Masson’s Trichrome staining revealed the absence of fibrosis in the sham group.

**Fig. 4:**
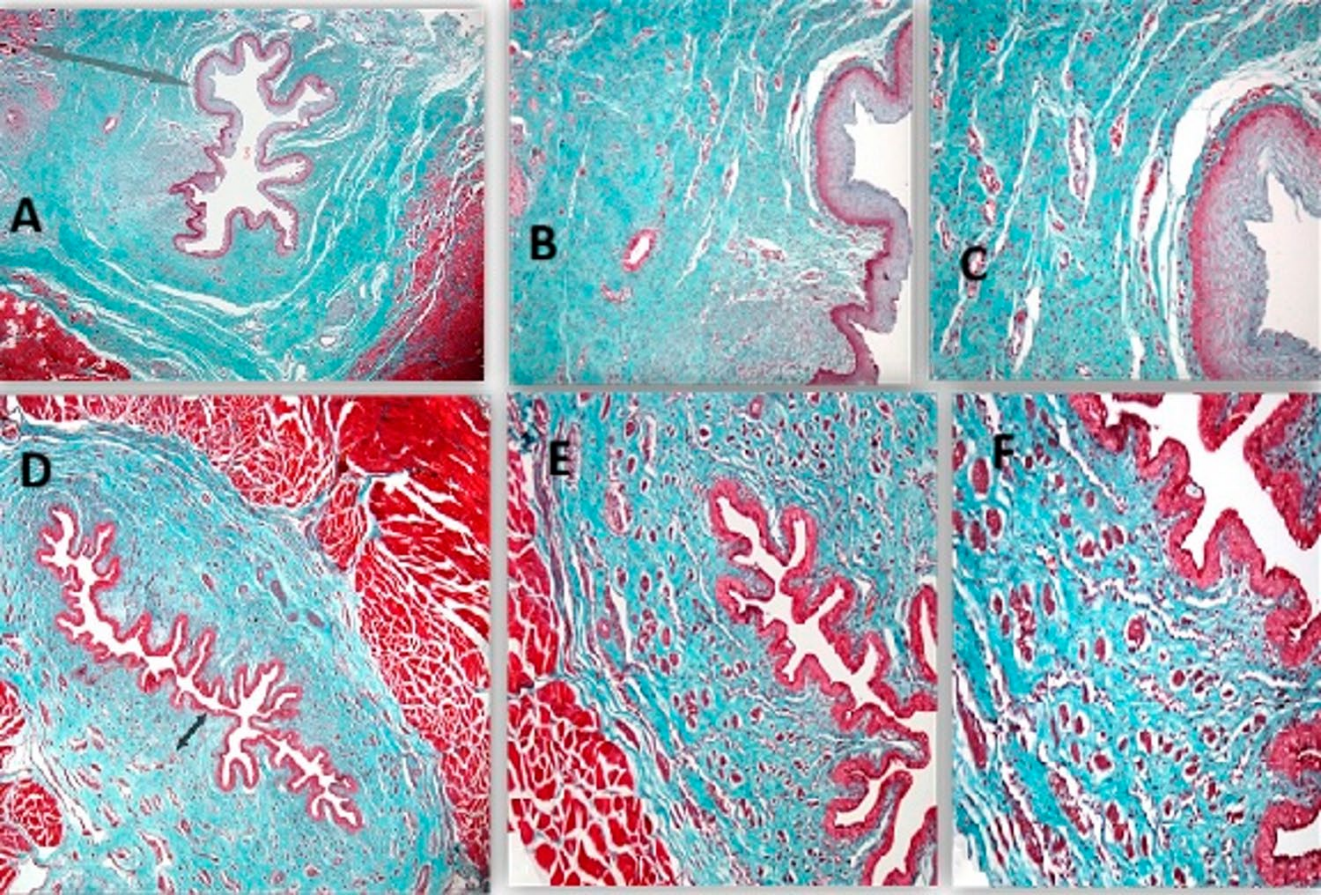
Representative pathological cross-sectional images of urethral tissue. Group 2. **a** Marked collagen deposition in the submucosal tissue (Masson trichrome, ×10). **B**, **C** Higher-magnification views demonstrating dense submucosal collagen accumulation (Masson trichrome, ×25 and ×40, respectively) Group 3: **D** Mild collagen deposition in the submucosal tissue (Masson trichrome, ×10). **E**, **F** Higher-magnification views demonstrating submucosal collagen deposition (Masson trichrome, ×25 and ×40, respectively)

In Group 2, which did not receive treatment, mild collagen accumulation and fibrosis were observed in one rabbit, moderate in four rabbits, and severe collagen accumulation and fibrosis extending from the submucosal tissue to the muscle layer in five rabbits. In Group 3, which received HBO therapy, fibrosis was not observed in three rabbits, while mild submucosal collagen accumulation was observed in five rabbits and moderate fibrosis was detected in only two rabbits (Fig. 4).

 When the groups were compared according to fibrosis scores, fibrosis scores in Group 2, which did not receive treatment, were found to be significantly higher than those in the HBO treatment group (*p* < 0.001). No pathological findings consistent with fibrosis were observed in the sham group (Table 1).

## Discussion

 To our knowledge, this is the first study in the literature to evaluate the effect of HBO therapy on the development of stricture following urethral injury in an experimental animal model. Furthermore, it demonstrates that HBO significantly reduces the development of US. US is a significant clinical problem that can lead to complications including urinary tract infection, scrotal-perineal abscess, acute urinary retention, prostatitis, bladder stones, epididymitis-orchitis. If left untreated, the condition can progress to renal failure [2–4]. The low long-term success rates of endoscopic treatments, the high risk of recurrence, and the fact that open surgical methods are difficult, costly and require expertise underscore the necessity for novel strategies aimed at preventing stricture development from the outset or modifying the fibrotic response [3, 4].

 In the course of the studies, experimental US models were created in rats and rabbits using different techniques [9–15]. While the creation of strictures using the blind technique and the difficulty of endoscopic verification represent significant limitations in rat models, the ability to induce controlled damage endoscopically in rabbit models and subsequently perform objective evaluation using urethrography is a major advantage. Consequently, the present study is founded upon the utilisation of the rabbit model, a model that has been standardised endoscopically and has been demonstrated to be reliable in the extant literature. In the context of urethral wound healing, the stabilization of the fibrotic response is a critical milestone. While the early phases are dominated by inflammatory cell infiltration, the transition to organized collagen maturation and myofibroblast-induced contraction typically reaches a measurable peak by the fourth week. Our selection of a 28-day follow-up is not only consistent with the physiological timeline of chronic scar formation in luminal organs but also aligns with the established protocols of previous high-impact experimental models, thereby facilitating a reliable comparison of treatment efficacy [12, 13].

 The fundamental pathological processes in urethral stricture development are spongiofibrosis and collagen accumulation; collagen concentration has been reported to increase by approximately 32 per cent in stricture segments [14]. Concurrently, the majority of medical treatment studies have concentrated on the reduction of fibroblast proliferation, collagen synthesis and wound contraction. A range of studies have utilised a herbal preparation known as Sairei-to, incorporating triamcinolone, halofuginone, mitomycin C, halofuginone, tadalafil, botulinum toxin A, dexpanthenol, and erythropoietin, with the objective of mitigating stenosis development or the fibrotic response. The outcomes of these studies have yielded varying degrees of success [9–11, 14–19]. Nevertheless, these studies have not yet resulted in the formulation of a standard medical protocol for clinical practice. This situation demonstrates that US remains an area that is primarily managed by surgical/endoscopic methods and is not sufficiently resolved from a medical perspective.

 The delicate equilibrium between epithelialisation and myofibroblast-induced contraction is pivotal in the development of post-injury stricture. In instances where epithelialisation is delayed, there is an observed increase in myofibroblast activity, leading to augmented accumulation of granulation tissue and collagen. This, in turn, results in the inevitable narrowing of the lumen [20, 21]. Consequently, the acceleration of wound healing and the enhancement of oxygen delivery to hypoxic tissues can be considered rational objectives that have the potential to reduce scar formation. While tissue pO₂ levels are limited to 10–30 mmHg in many wounds, levels of 50–100 mmHg are required for optimal healing [5, 6, 22]. It has been demonstrated that HBO therapy increases tissue oxygenation by exceeding this physiological threshold, stimulates the release of growth factors (VEGF, PDGF, HIF-1/2, etc.) via reactive oxygen and nitrogen products, accelerates angiogenesis, and increases bactericidal activity [5, 23]. The multifaceted effects of HBO render it a unique modality that plays a role in virtually every phase of wound healing.

The aetiology of US is multifactorial, with a strong predilection for underlying conditions including, but not limited to, trauma, infection, endoscopic procedures and ischaemia. HBO therapy has been demonstrated to enhance blood flow and accelerate wound closure in cases involving ischaemia, hyperglycaemia, infection and trauma. Additionally, it has been observed to promote fracture healing in models of ischaemia and hyperglycaemia [24, 25]. The positive effects of HBO on wound healing have also been demonstrated in different organ systems. In the domain of urology, its indications are restricted to specific pathological conditions, including Fournier’s gangrene, radiation cystitis, and interstitial cystitis [26–30]. However, it has been demonstrated that HBO can enhance the efficacy of buccal grafts in hypospadias surgery, leading to a reduction in the incidence of fistula and stricture [31, 32]; Additionally, HBO has been shown to expedite epithelialisation and decrease stenosis rates in tracheal resection and anastomosis [33, 34]; Furthermore, HBO has been observed to mitigate ulceration, inflammation, mortality, and stricture development in oesophageal injury models [35], underscoring its substantial healing potential in lumen-containing organs susceptible to fibrosis. The observation that the development of fibrosis-related strictures in luminal organs such as the oesophagus and urethra exhibits a comparable pathophysiology suggests that these data may also have translational value for US.

 There is a paucity of consensus in the extant literature regarding the standardisation of HBO therapy in terms of duration, pressure, and number of sessions. Ren et al. administered HBO at 2 ATA for 90 min over periods ranging from 7 to 28 days in a hypertrophic scar model in rabbit ears and reported significant benefits, particularly with the 21- and 28-day treatment protocols [36]. In the present study, a 21-day treatment regimen was applied, comprising 90-minute sessions at 2 ATA, in accordance with the protocol referenced in the literature. This protocol has been shown to be effective in the rabbit model.

The findings of our study demonstrate that subjects in the group with stricture formation and no treatment exhibited significant fibrosis and substantial collagen accumulation histopathologically, as well as severe lumen narrowing endoscopically and urethrographically. Conversely, the group receiving HBO therapy exhibited a significantly lower fibrosis score, a wider stricture diameter, and a lower percentage of narrowing. The absence of a significant difference in the diameters of normal urethral segments between groups supports the hypothesis that the detected changes are directly related to the induced injury and the applied treatment. In this regard, the present study offers preliminary experimental evidence that HBO can effectively mitigate stricture development by modulating the fibrotic response subsequent to urethral injury.

Taken together, these results suggest that HBO may represent a promising adjuvant option for the future management of US, particularly in settings where recurrent disease remains the major therapeutic challenge. As recurrence constitutes the most significant problem in stricture treatment, the use of HBO as a perioperative supportive treatment after internal urethrotomy or reconstructive surgery may help attenuate fibrotic remodelling and thereby reduce recurrence rates. From a translational perspective, our experimental findings also raise the hypothesis that early initiation of HBO soon after traumatic urethral injury or clinically relevant iatrogenic mucosal damage (e.g. difficult catheterisation, TUR-P, urethroscopy, prolonged catheterisation) could mitigate the extent of spongiofibrosis and decrease the likelihood or severity of subsequent stricture formation in selected high-risk patients. In addition, in individuals with recurrent strictures undergoing repeated direct visual internal urethrotomy, perioperative HBO protocols might modulate wound healing dynamics around the urethral lumen and potentially improve long-term patency. Any potential clinical application, however, would require carefully designed prospective clinical trials to define the optimal timing, pressure, session duration and total number of HBO treatments, as well as to identify appropriate patient subgroups and contraindications, before routine use in daily practice can be recommended. From a clinical perspective, it is important to emphasize that while radiological and endoscopic success is vital, urethral stricture is ultimately a functional disease. Therefore, future translational and clinical studies should not rely solely on anatomical outcomes but should also incorporate functional parameters, including uroflowmetry, post-void residual volume, and validated patient-reported outcome measures, to more accurately capture clinical relevance and quality-of-life impact.

 By demonstrating in a standardised animal model that HBO can significantly reduce radiological urethral narrowing and histopathological fibrosis, the present study helps to fill an important gap in the literature and provides preclinical support for further translational research on the role of HBO in the prevention and adjuvant treatment of US.

This study has several limitations that should be acknowledged. First, the follow-up period was limited to 28 days; therefore, late tissue remodelling and long-term stricture recurrence could not be adequately assessed. Second, we used a single HBO protocol (2 ATA, 90 min/day for 21 days), and no dose–response analysis was performed, which precludes conclusions about the optimal pressure, duration, or total number of sessions. Third, our endpoints were predominantly morphological, based on radiological narrowing and histopathological fibrosis scores; functional assessments such as uroflowmetry or urodynamic measurements were not undertaken. Acknowledging that urethral stricture is a functional disorder, the absence of these measurements limits our insight into the physiological impact of the treatment. Future studies should prioritize these functional parameters, alongside post-void residual volume and clinical symptom scores, to better establish the clinical utility of HBO. Fourth, we did not include a sham-HBO or “injury + compressed air” control group, which would have allowed us to fully disentangle the specific effects of oxygen from those related to chamber pressure or environmental factors. Fifth, we did not investigate molecular markers of inflammation, angiogenesis, or fibrosis (e.g. cytokine profiles, growth factors, collagen subtypes), which limits our ability to elucidate the precise mechanistic pathways through which HBO modulates urethral wound healing. Finally, a formal priori sample size calculation was not performed, and the number of animals per group was determined based on ethical considerations and previous experimental models; as a result, the statistical power and generalisability of our findings may be limited and should be confirmed in larger, independent studies.

## Conclusion

 In conclusion, this experimental study shows that hyperbaric oxygen therapy can attenuate fibrosis and reduce the severity of urethral stricture after urethral trauma, consistent with its multifaceted effects on wound healing. These findings suggest that HBO may be a promising adjuvant strategy for the prevention of urethral stricture and the reduction of its recurrence. However, translation into routine clinical practice will require well-designed clinical trials with larger cohorts, appropriate control groups, long-term follow-up, and formal assessment of cost-effectiveness.

## Data Availability

All datasets used and analyzed during the current study are available from the corresponding author upon reasonable request.

## Abbreviations

ATA: Atmosphere absolute
CO: Carbon monoxide
EPO: Erythropoietin
H&E: Hematoxylin–eosin
HIF-1/2: Hypoxia-Inducible factor-1/2
HBO: Hyperbaric oxygen therapy
NaOH: Sodium hydroxide
PDE-5: Phosphodiesterase type 5
PDGF: Platelet-derived growth factor
SPSS: Statistical package for the social sciences
TUR-P: Transurethral resection of the prostate
US: Urethral stricture
VEGF: Vascular endothelial growth factor
