# Correction: Hyperbaric oxygen therapy attenuates urethral stricture formation after urethral injury: an experimental rabbit study

**DOI:** 10.1007/s00345-026-06382-5

**Published:** 2026-05-11

**Authors:** Hasan Danış, Alpaslan Yüksel, Dursun Baba, Yusuf Şenoğlu, Arda Taşkın Taşkıran, Ekrem Başaran, Ali Tekin, Sinem Kantarcloélu

**Affiliations:** 1 Department of Urology, Duzce Ataturk State Hospital, Duzce, Türkiye; 2 Department of Urology, Medeniyet University Prof. Dr. Süleyman Yalçın Hospital, İstanbul, Türkiye; 3 Department of Urology, Duzce University School of Medicine, Duzce, Türkiye; 4 Department of Urology, Marmara University School of Medicine, Istanbul, Türkiye; 5 Department of Urology, Acıbadem University Atakent Hospital, İstanbul, Türkiye; 6 Department of Pathology, T.C. Ministry of Health, Ballkesir, Türkiye

**Correction: World Journal of Urology (2026) 44:220** 10.1007/s00345-026-06323-2

In the original version of the article, the following author should be added as 8th author to the article.

Sinem Kantarcloélu.

Department of Pathology, T.C. Ministry of Health, Ballkesir.

The original article has been corrected.

